# Frequency and Duration of Diagnostic Delays Associated with Coccidioidomycosis and Risk Factors for Missed Diagnoses, United States

**DOI:** 10.3201/eid3205.251421

**Published:** 2026-05

**Authors:** Desmond D. Barber, Alan T. Arakkal, George R. Thompson, John W. Baddley, Joe E. Cavanaugh, Aaron C. Miller, Philip M. Polgreen

**Affiliations:** University of Iowa College of Medicine, Iowa City, Iowa, USA (D.D. Barber, A.T. Arakkal, J.E. Cavanaugh, A.C. Miller, P.M. Polgreen); University of California, Davis, California, USA (G.R. Thompson); Johns Hopkins University, Baltimore, Maryland, USA (J.W. Baddley)

**Keywords:** fungi, diagnosis, delay, coccidioidomycosis, United States

## Abstract

Diagnosis of coccidioidomycosis is challenging and requires a high index of clinical suspicion. We estimated the incidence and duration of, and risk factors associated with, diagnostic delays and missed opportunities in the diagnosis of coccidioidomycosis. We conducted a retrospective analysis of health insurance claims data in the United States during 2001–2022 included in the Merative MarketScan Databases. Using a case-crossover design and a bootstrapping approach, we estimated the number of excess visits for coccidioidomycosis-related symptoms before diagnosis. We also evaluated potential factors associated with delay. We estimated that almost 60% of patients experienced >1 missed opportunity for diagnosis; the average diagnostic delay was 29.69 (95% CI 28.25–31.18) days. Missed opportunities were predominantly observed in outpatient settings (73%) and were significantly associated with older age, rural residence, underlying pulmonary conditions, and prescriptions for antibiotics or inhalers. Diagnostic delays for coccidioidomycosis are common, and addressing such delays could improve clinical outcomes.

Coccidioidomycosis is an infectious disease caused by dimorphic fungi of the genus *Coccidioides* (*1*). Infections are caused by *C. immitis* and *C. posadasii,* and the clinical manifestations for both species are similar (*2*). In the United States, most cases occur in Arizona and California (*3*), but sporadic cases also occur in parts of Texas, New Mexico, Utah, and Nevada (*4*). The geographic range of infection might be more expansive than previously thought, and the range of the organisms might be increasing both northward and eastward within the continental United States (*5*). Furthermore, the incidence of coccidioidomycosis also appears to be increasing (*6*).

*Coccidioides* spp. grow as mold in the environment, and inhalation of airborne arthroconidia is the most common route of infection. Approximately 60% of cases are asymptomatic (*7*). Among symptomatic cases, common symptoms consist of cough, fever, chills, shortness of breath, chest discomfort, and fatigue, producing a clinical picture that closely mimics viral respiratory infection or pneumonia (*8*,*9*). Although primary pulmonary disease is most common, dissemination to extrapulmonary sites can occur through hematogenous or lymphatic spread, and the skin, bones, joints, and central nervous system are the most frequent targets (*10*). Overall, disseminated disease is rare (≈1% of cases) (*11*), but its risk is substantially elevated in immunocompromised persons (*12*).

Diagnosis of coccidioidomycosis is challenging and requires a high index of clinical suspicion, because the symptoms, physical examination findings, and radiographic findings for the disease are nonspecific. Given the clinical overlap of signs and symptoms with bacterial pneumonia or other respiratory symptoms, patients with coccidioidomycosis are frequently treated with multiple courses of antibiotics before diagnosis, even in regions where the disease is relatively common (*13*–*16*). Diagnosis is further complicated by the limitations of available testing modalities. Serologic testing for antibodies offers relatively high sensitivity (*17*) but can be negative early in the course of the disease, and ordering serologic testing requires suspicion of coccidioidomycosis on the part of the clinician. Understanding the timeliness of diagnosis is key not only for improving clinical care but also for public health response and disease surveillence. Delays in diagnosis can obscure the apparent timeline of infection, leading to bias in surveillance, outbreak detection, and source attribution.

Because of the potential of diagnostic delays for coccidioidomycosis, better characterization of the incidence and factors associated with those delays is urgently needed. Some studies of coccidioidomycosis-related diagnostic delays have been conducted (*15*,*18*,*19*), but larger studies that include risk factors for delays are needed. The purpose of this study was to use a large database of commercial insurance claims to estimate the incidence of delays in diagnosing coccidioidomycosis, estimate the average length of diagnostic delays, and identify potential factors associated with delayed diagnosis of coccidioidomycosis, including use of unnecessary antibiotics.

## Methods

### Data Source and Study Population

We conducted a retrospective study using longitudinal health insurance claims from the Merative MarketScan Research Databases, including the Commercial and Medicare databases from 2001–2022 and the Multi-State Medicaid databases from 2014–2021. Those data represent one of the largest databases of commercial insurance claims in the United States and contain records from inpatient, outpatient, and emergency department encounters, along with outpatient prescription medications. Over the study period, the Commercial and Medicare databases represented ≈29 million daily enrollees, whereas the Medicaid database represented ≈11 million daily enrollees, on average.

We identified patients with coccidioidomycosis using diagnosis code 114.X from the International Classification of Diseases, 9th Revision, Clinical Modification (ICD-9-CM), and code B38.XX from the International Classification of Diseases, 10th Revision, Clinical Modification (ICD-10-CM). We identified the index coccidioidomycosis diagnosis as the first healthcare visit during which coccidioidomycosis was diagnosed. Patients were required to be continuously enrolled for >1 year before the index coccidioidomycosis diagnosis; thus, we excluded patients who received a diagnosis of coccidioidomycosis in 2001.

### Statistical Analysis

We identified all potential missed diagnostic opportunities by evaluating healthcare visits before the index coccidioidomycosis diagnosis in which potential signs or symptoms of coccidioidomycosis were present. We reviewed all ICD-9-CM and ICD-10-CM diagnosis codes recorded during healthcare visits in the year before the index coccidioidomycosis diagnosis to identify visits in which the patient displayed signs or symptoms of coccidioidomycosis, visits in which tests were ordered for conditions with similar manifestations as coccidioidomycosis, or visits in which conditions were diagnosed that have similar manifestations to coccidioidomycosis. Codes meeting 1 of those criteria were deemed clinically plausible as potential evidence of underlying coccidioidomycosis and defined as symptomatically similar diagnoses (SSDs). We used the complete set of SSDs and corresponding ICD-9-CM and ICD-10-CM codes to identify potential missed diagnostic opportunities (Appendix Table 1).

Missed opportunities to diagnose coccidioidomycosis are not directly observable, because signs or symptoms occurring before diagnosis (e.g., cough, fever, fatigue) might be coincidental and caused by other diseases with similar symptomology (e.g., respiratory infection). Thus, to estimate the actual number of missed opportunities, we used the 2-step process described next, which has been used in multiple studies to evaluate diagnostic delays (*20*–*25*). Extensive details of this methodological approach can be found in Miller et al. (*26*).

#### Step 1—Estimating the Number of Missed Opportunities. 

We began by implementing a type of case-crossover design to calculate the excess number of SSD visits each day before the coccidioidomycosis diagnosis. We specified a case period of 13 weeks (91 days) before the coccidioidomycosis diagnosis. That period is referred to as the diagnostic opportunity window, defined as the time before diagnosis when diagnostic opportunities might occur. The period from 92–365 days before the coccidioidomycosis diagnosis represents the crossover (control) period for each enrollee. We selected the 13-week cutoff as the point in time when the frequency of SSD-related visits began to increase (Appendix Figure 1). We also conducted a sensitivity analysis evaluating alternative crossover periods (Appendix Table 2).

To estimate the expected number of SSD visits each day before diagnosis, we computed the number of patients with an SSD-associated visit each day during the crossover period (92–365 days before diagnosis) and fit a quadratic time trend to those daily visit counts, including an additive effect for day of week to capture weekly periodicity. We then extrapolated that trend forward into the diagnostic opportunity window (i.e., 1–91 days before diagnosis) to estimate the expected number of SSD visits. Finally, we computed the number of missed diagnostic opportunities each day before diagnosis as the difference between the observed and expected number of SSD visits (i.e., the excess number of SSD visits) during the diagnostic opportunity window.

#### Step 2—Estimating Diagnostic Delay Metrics and Potential Risk Factors

We used a bootstrapping approach to estimate patient-level measures for the frequency and duration of and potential risk factors for diagnostic delays (*27*). First, we randomly draw individual patient visits representing a missed opportunity on a given day using the computed number of missed opportunities from the case-crossover analysis (described previously). Using those selected visits, we calculated the number of patients experiencing a missed opportunity, the number of missed opportunities per patient, the number of missed opportunities by healthcare setting, and duration of diagnostic delay, then evaluated the risk-factor models described next.

We implemented the bootstrapping procedure using the following 4-step process: draw a bootstrapped sample of patients with replacement, implement the case-crossover analysis computing the number of missed opportunities each day before diagnosis, randomly draw which individual patient visits represented a missed opportunity (using an uncorrelated algorithm [*27*]), and calculate delay metrics and evaluate the risk-factor models (outlined next). We repeated steps 1–2 100 times, generating 100 bootstrapped samples. Then, for each bootstrapped sample, we repeated steps 3–4 100 times. That process resulted in a total of 10,000 trials. We aggregated results across all trials and reported the median of the bootstrapped estimates along with the 95% percentile-based CI.

#### Risk Factors for Experiencing a Missed Opportunity 

We also conducted an exploratory analysis to identify possible risk factors associated with missed diagnostic opportunities. We evaluated a patient-level logistic regression model to estimate the odds of a patient experiencing a missed opportunity as a function of patient and clinical characteristics. For each simulation trial, we assigned patients selected as having a missed opportunity an outcome value of 1 (i.e., missed opportunity) and those not selected a value of 0 (i.e., no missed opportunity). Patient demographics evaluated included patient age group (categorized into <18, 18–34, 35–44, 45–54, 55–64, and >65 years), sex, and rurality. We created an indicator for rurality if the patient visited a rural health clinic (using the place of service variable on a claim record). In addition, we considered patients’ clinical history of underlying pulmonary conditions that might induce cognitive bias into the diagnostic process. Specifically, we included indicators for underlying history of asthma, chronic obstructive pulmonary disease (COPD), and chest imaging (radiograph or computed tomography scan). Furthermore, we evaluated whether treatments for potential symptoms of coccidioidomycosis affected delays by including indicators for respiratory antibiotics and inhalers prescribed during the diagnostic opportunity window. We also included month and year of the index coccidioidomycosis diagnosis as categorical variables.

We conducted all statistical analyses using R version 3.5.1 (The R Project for Statistical Computing, https://www.r-project.org) and implemented the bootstrapping procedure using the delaySim package (https://github.com/aarmiller/delaySim), which we maintain. All of the code used for identifying cases, along with diagnosis, procedure, and medication codes, and for conducting all statistical analysis can be found at the GitHub repository (https://github.com/aarmiller/delay_diagnosis/publications/cocci). This study uses completely deidentified observational data and is thus classified as non–human subjects research according to the National Institutes of Health under category 4.

### Secondary Analyses

We conducted 2 secondary analyses to evaluate geographic variability in delays and to evaluate potential excess antibiotic use as the result of delays. To evaluate differences in delay characteristics between patients residing in Arizona and those living elsewhere, we stratified the study population accordingly, repeated each analysis, and compared the resulting delay metrics of interest. To evaluate whether delays were potentially linked to excess antibiotic use, we identified all outpatient antibiotic prescriptions occurring in the year before diagnosis and compared that trend to SSD-associated visits.

## Results

We identified 44,292 cases of coccidioidomycosis (43,210 from the Commercial and Medicare databases and 1,082 from the Medicaid database) during 2001–2022. Of those cases, 26,905 met the inclusion criteria of being continuously enrolled for >1 year before their index diagnosis (26,379 from Commercial and Medicare and 526 from Medicaid). The median age of patients in the cohort was 52.0 years; 51.5% of the cohort patients were female and 48.5% were male (Table 1). Most patients were identified in the Commercial claims database (80.2%), followed by the Medicare (17.9%) and Multi-State Medicaid (2.0%) databases.

**Table 1:** Baseline characteristics of study population in study of frequency and duration of diagnostic delays associated with coccidioidomycosis and risk factors for missed diagnoses, United States*

Characteristic	Value
Age at diagnosis, y	
<18	1,686 (6.3)
18–34	3,531 (13.1)
35–44	4,026 (15.0)
45–54	5,581 (20.7)
55–64	6,930 (25.8)
>65	5,151 (19.1)
Mean (+SD)	50.1 (18.4)
Median (IQR)	52.0 (23.0)
Sex	
M	13,056 (48.5)
F	13,849 (51.5)
Database source	
Commercial	21,573 (80.2)
Medicare	4,806 (17.9)
Medicaid	526 (2.0)
Enrollment time before index diagnosis, y	
<2	7,177 (26.7)
<3	12,219 (45.4)
>3	14,686 (54.6)
Mean (+SD)	4.4 (3.4)
Median (IQR)	3.3 (3.9)

*Values are no. (%) patients except as indicated. IQR, interquartile range.

**Figure 1:**
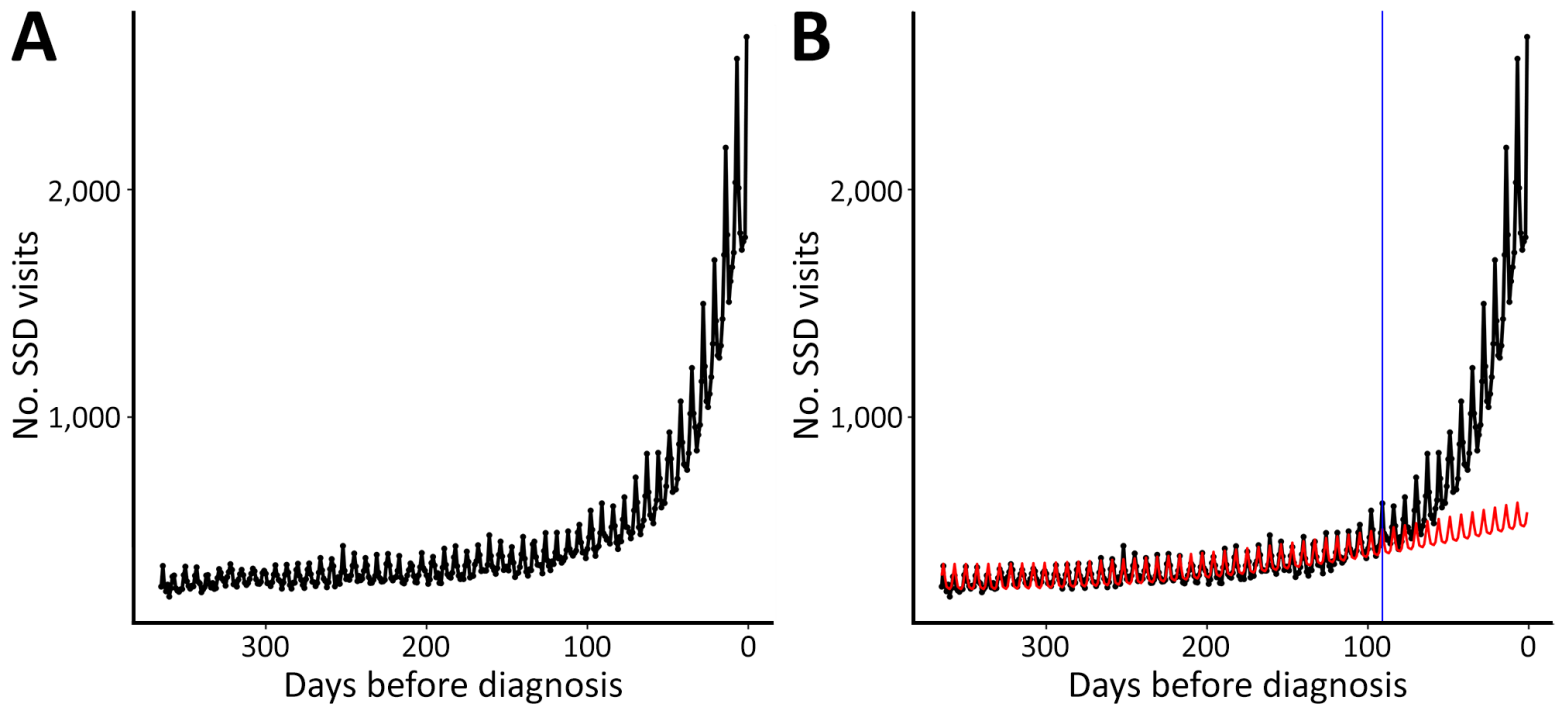
Trends in SSD-related healthcare visits in the 365 days before index coccidioidomycosis diagnosis in study of frequency and duration of diagnostic delays associated with coccidioidomycosis and risk factors for missed diagnoses, United States. A) Raw SSD visit counts for each day before the index coccidioidomycosis diagnosis. B) Expected trendline (in red) estimated from visits between 92–365 days before the index coccidioidomycosis diagnosis; vertical blue line indicates start of the diagnostic opportunity window, beginning 91 days before the index diagnosis. Estimated number of missed diagnostic opportunities is depicted as the area between the observed line (in black) and the expected trend (in red) during the diagnostic opportunity window (region to the right of the blue line). SSD, symptomatically similar diagnosis.

In the year before the index coccidioidomycosis diagnosis, 26,398 (98.1%) patients had >1 healthcare visit for any reason, and 23,191 (86.2%) patients had >1 SSD visit. A total of 177,679 SSD-associated visit days were noted in the 365 days before the index diagnosis. The frequency of SSD-related visits increased substantially beginning ≈100 days before the index diagnosis (Figure 1, panel A).

**Figure 2:**
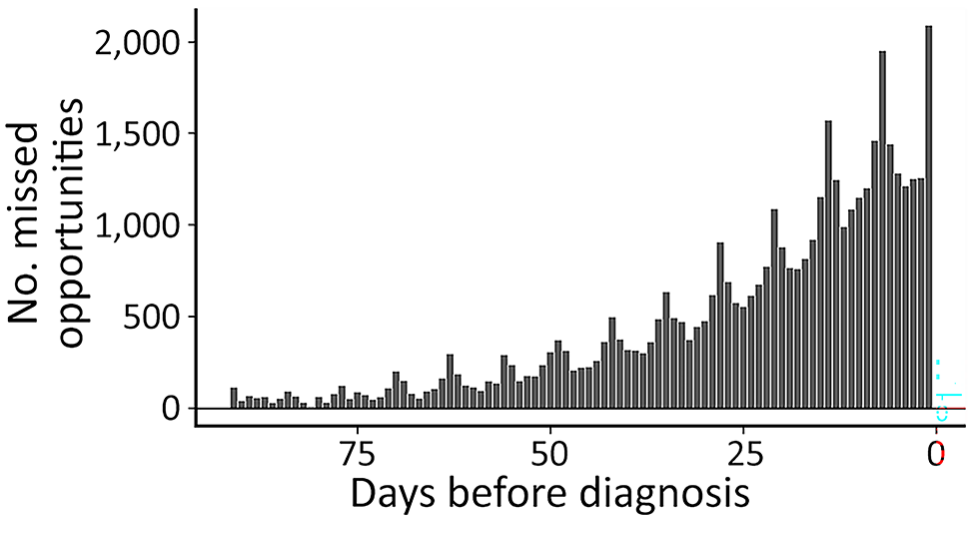
Estimated number of missed diagnostic opportunities each day before the index coccidioidomycosis diagnosis in study of frequency and duration of diagnostic delays associated with coccidioidomycosis and risk factors for missed diagnoses, United States. Estimates are based on the excess number of symptomatically similar diagnosis–associated healthcare visits, computed as the difference between the observed and expected values in Figure 1.

During the 13-week diagnostic opportunity window, 88,130 total SSD visit days from 19,670 (73.1%) patients occurred, representing potential missed opportunities. We plotted the expected trend (in red) of SSD-related visits, estimated on the basis of visits during the crossover period from 92–365 days before diagnosis (Figure 1, panel B). We also plotted the distribution of the estimated number of missed diagnostic opportunities each day before the index diagnosis (Figure 2).

On the basis of the bootstrapping approach, we estimated that 43,338 (95% CI 40,566–46,125) of total SSD visit days during the diagnostic opportunity window represented actual missed diagnostic opportunities (Table 2). Approximately 73.2% (95% CI 72.4%–74.1%) of missed opportunities occurred in an outpatient setting, 18.6% (95% CI 17.8%–19.5%) in inpatient settings, 7.5% (95% CI 7.2%–7.7%) in emergency department settings, and 0.7% (95% CI 0.6%–0.7%) in observational stay settings.

**Table 2:** Index diagnosis visits and simulation results for missed diagnostic opportunities by setting in study of frequency and duration of diagnostic delays associated with coccidioidomycosis and risk factors for missed diagnoses, United States*

Setting	No. potential missed opportunity visit days	Index diagnosis visits†			Potential missed opportunities‡	
		No. index visits	% Of all index visits		No. missed opportunities (95% CI)	% Of all missed opportunities (95% CI)
Outpatient	71,392	25,237	84.4%		35,435 (33,277–37,741)	73.2% (72.4%–74.1%)
Emergency department	6,985	1,230	4.1%		3,607 (3,330–3,838)	7.5% (7.2%–7.7%)
Observational stay	626	128	0.4%		318 (271–365)	0.7% (0.6%–0.7%)
Inpatient	18,897	3,314	11.1%		9,024 (8,280–9,834)	18.6% (17.8%–19.5%)
Inpatient visit§	3,169	3,314	11.1%		2,654 (2,532–2,784)	NC§

*NC, not calculated. †Multiple settings may be associated with an index visit date (i.e., we are unable to order the timing of visits from claims on the same day); thus, the total number exceeds the number of individuals in the study population. ‡Multiple settings may occur on a given visit day (i.e., we are unable to order the timing of visits from claims on the same day); thus, the total number exceeds the total estimated potential missed diagnostic opportunities. §Inpatient visits represent >1 days admitted to an inpatient facility. Our analysis of missed diagnostic opportunities is based on visit days in which the patient received a symptomatically similar diagnosis on a given day. We can aggregate potential missed opportunities, index diagnoses, and estimated missed opportunities from the bootstrapping approach into the overall inpatient stay in which they occurred. However, the denominator for computing the percent of missed opportunities that occurred in each setting represents the total number of visit days representing a missed opportunity; thus, a value for aggregate inpatient visits is not computed.

Approximately 59.7% (95% CI 58.3%–61.1%) of patients experienced >1 missed diagnostic opportunity (Table 3). Of the patients who experienced >1 missed opportunity, they experienced on average 2.70 (95% CI 2.58–2.81) visits representing missed opportunities and had an average diagnostic delay duration of 29.69 days (95% CI 28.25–31.18). An estimated 24.7% (95% CI 22.9%–26.7%) of patients experienced a diagnostic delay duration that lasted >30 days.

**Table 3:** Simulation results for number and duration of delayed visits per patient in study of frequency and duration of diagnostic delays associated with coccidioidomycosis and risk factors for missed diagnoses, United States *

Metric	Value	95% CI
No. (%) missed opportunities per patient		
≥1	16,061 (59.7%)	15,685–16,445 (58.3%–61.1%)
≥2	9,770 (36.3%)	9,312–10,238 (34.6%–38.1%)
≥3	5,824 (21.6%)	5,408–6,281 (20.1%–23.3%)
≥4	3,491 (13.0%)	3,159–3,849 (11.7%–14.3%)
≥5	2,183 (8.1%)	1,935–2,447 (7.2%–9.1%)
Mean	2.70	2.58–2.81
Median	2.00	2.00–2.00
Duration of delayed visits, d		
≥1	16,061 (59.7%)	15,685–16,445 (58.3%–61.1%)
≥3	15,113 (56.2%)	14,724–15,516 (54.7%–57.7%)
≥7	13,780 (51.2%)	13,376–14,213 (49.7%–52.8%)
≥10	12,453 (46.3%)	12,027–12,895 (44.7%–47.9%)
≥14	11,187 (41.6%)	10,738–11,625 (39.9%–43.2%)
≥21	9,050 (33.6%)	8,570–9,534 (31.9%–35.4%)
≥30	6,650 (24.7%)	6,169–7,177 (22.9%–26.7%)
≥45	3,930 (14.6%)	3,458–4,447 (12.9%–16.5%)
≥60	2,150 (8.0%)	1,781–2,580 (6.6%–9.6%)
≥90	153 (0.6%)	82–229 (0.3%–0.9%)
Mean delay duration, d	29.69	28.25–31.18
Median delay duration, d	24.05	23.00–26.00

*The distribution and mean number of potential missed diagnostic opportunities each patient experienced along with the distribution and mean and median duration of diagnostic delays (in days) are presented. Potential missed diagnostic opportunities represent healthcare visits in which sign/symptoms were present, but coccidioidomycosis was not diagnosed. Delay duration was defined as the time between the earliest potential missed diagnostic opportunity a patient experienced and their index diagnosis.

Patients with Medicare (odds ratio [OR] 0.61 [95% CI 0.47–0.78]) or Medicaid (OR 0.61 [95% CI 0.48–0.77]) were less likely to experience a delay (i.e., experience >1 missed diagnostic opportunity) than were patients with commercial insurance (Table 4; Appendix Table 3). Patients >65 years of age were more likely to experience a delay than were patients <18 years of age (OR 1.48 [95% CI 1.15–1.98]). Patients in rural locations were slightly more likely to experience a delay (OR 1.27 [95% CI 1.04–1.60]). Patients with underlying history of pulmonary conditions or related procedures were more likely to experience a diagnostic delay (i.e., asthma [OR 1.34 (95% CI 1.18–1.49)], COPD [OR 1.56 (95% CI 1.36–1.80)], chest computed tomography [OR 1.42 (95% CI 1.26–1.61)], and chest radiography [OR 1.21 (95% CI 1.12–1.30)]). Finally, receipt of medications to treat potential symptoms of coccidioidomycosis during the diagnostic opportunity window was positively associated with the likelihood of experiencing a diagnostic delay (i.e., antibiotics [OR 3.42 (95% CI 3.18–3.69)] and inhalers [OR 2.64 (95% CI 2.34–2.99)]).

**Table 4:** Results of exploratory logistic regression model in study of frequency and duration of diagnostic delays associated with coccidioidomycosis and risk factors for missed diagnoses, United States*

Potential risk factor	Odds ratio (95% CI)
Database source	
Commercial	Referent
Medicare	0.61 (0.47–0.78)
Medicaid	0.61 (0.48–0.77)
Age group, y	
<18	Referent
18–34	0.89 (0.77–1.00)
35–44	0.94 (0.82–1.08)
45–54	0.94 (0.81–1.06)
55–64	0.93 (0.82–1.05)
>65	1.48 (1.15–1.98)
Female sex	0.99 (0.93–1.05)
Rural location	1.27 (1.04–1.60)
Asthma diagnosis before delay window	1.34 (1.18–1.49)
COPD diagnosis before delay window	1.56 (1.36–1.80)
Chest CT before delay window	1.42 (1.26–1.61)
Chest radiography before delay window	1.21 (1.12–1.30)
Respiratory antibiotic prescriptions filled during delay window	3.42 (3.18–3.69)
Inhaler prescriptions filled during delay window	2.64 (2.34–2.99)

*Odds ratios corresponding to the odds of a patient experiencing a diagnostic delay are aggregated across bootstrap trials. The mean odds ratio along with 95% CI are reported across trials for each potential risk factor. See Appendix Table 3 for effects associated with month and year. COPD, chronic obstructive pulmonary disorder; CT, computed tomography.

We observed similar patient-level delay metrics for patients residing in Arizona and patients not residing in Arizona (Appendix Table 4). However, a greater proportion of patients in the Arizona cohort experienced >1 missed diagnostic opportunity than did patients in the non-Arizona cohort (69.6% vs. 55.5%).

**Figure 3:**
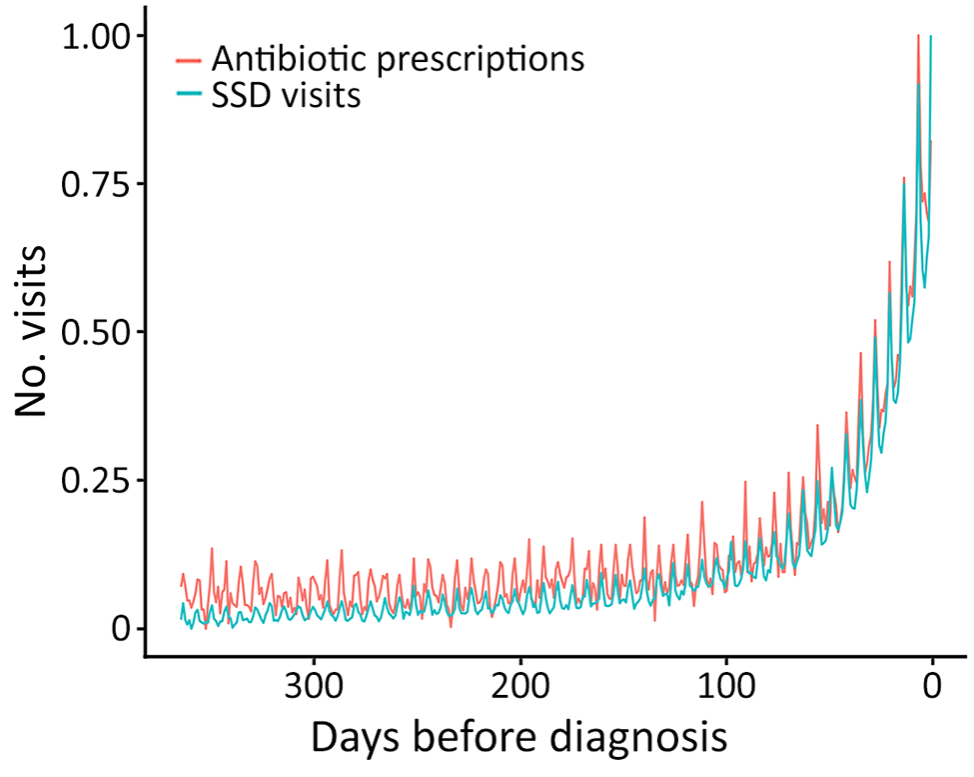
Number of patients with an outpatient antibiotic prescription or SSD visit each day before the index coccidioidomycosis diagnosis in study of frequency and duration of diagnostic delays associated with coccidioidomycosis and risk factors for missed diagnoses, United States**.** For comparison, both series have been minimum–maximum normalized between 0 and 1 to enable comparison of their trends; see Appendix Figure2 or raw counts of antibiotics before diagnosis. The trend in SSD visits and outpatient antibiotics exhibit a nearly identical pattern before diagnosis, with a similar increase occurring around 90 days before diagnosis. Those trends suggest an increase in unnecessary antibiotics prescribed in response to undiagnosed coccidioidomycosis symptoms before diagnosis. SSD, symptomatically similar diagnosis.

The number of outpatient antibiotic prescriptions increased substantially beginning nearly 90 days before diagnosis (Appendix Figure 2). The trend in antibiotic prescriptions mirrored the trend in SSD visits, suggesting that SSD visits attributable to diagnostic delays were associated with excess antibiotic prescriptions (Figure 3).

## Discussion

We found that many patients who ultimately received a diagnosis of coccidioidomycosis experienced multiple healthcare encounters before diagnosis, and many of those visits represented potential missed opportunities for earlier diagnosis. Specifically, 60% of patients experienced >1 missed opportunity; among those persons, we estimated an average of 2.70 visits per patient to be potential missed opportunities, and ≈73% occurred in the outpatient setting. We estimated that the mean diagnostic delay was ≈30 days (median 24 days). In addition, elderly patients, patients living in rural areas, and those with underlying respiratory conditions were more likely to experience a delay in diagnosis. Furthermore, prescription of antibiotics or inhalers during the delay window increased the odds of experiencing a missed opportunity to diagnose coccidioidomycosis.

Coccidioidomycosis, similar to other endemic fungal infections, is an infectious disease that is commonly associated with diagnostic delays (*28*). Overall, our results largely align with previous studies reporting diagnostic delays for coccidioidomycosis. For example, a study of health claims for 139 patients in a large health system based in Phoenix, Arizona, found that 46% of patients had a diagnostic delay >1 month in length (*19*). A study in 14 US states found that among 339 patients, a median of 38 days passed between first seeking care and diagnosis (*29*). However, those studies might have described longer delays because determining when symptoms began is often difficult. For example, Benedict et al. (*29*) reported 1 case in their series with an extremely long interval of 1,654 days (*29*). Moreover, symptoms caused by other conditions have often been attributed to coccidioidomycosis in previous study designs of diagnostic delays (*29*,*30*). We included a much larger sample size than previous studies and used a case-crossover and bootstrapping approach, enabling us to distinguish attributable visits from background healthcare use, providing more accurate estimates of missed diagnostic opportunities. Indeed, our finding that 59.7% of patients experienced a diagnostic delay directly contrasts with a recent study (*30*) that used the same data source and similar study population but reported that 71.3% of patients experienced a diagnostic delay. The difference in our findings is most likely attributable to the fact that the previous study did not account for an expected level of background healthcare utilization and, consequently, labeled every patient with potential signs or symptoms in the 90 days before diagnosis as a case of diagnostic delay.

We identified multiple factors associated with the odds of diagnostic delays for coccidioidomycosis. First, delays were more common in older patients (i.e., >65 years of age compared with persons <18 years of age), an unsurprising finding, given that older adults frequently demonstrate more vague or subtle signs and symptoms for infectious diseases (*31*,*32*). For example, older adults are substantially less likely to experience a fever than are younger patients (*33*). Second, patients living in rural areas were more likely to experience a missed opportunity to diagnose coccidioidomycosis. Diagnostic delays among rural populations might be partially attributed to lack of access to medical care. Rural patients are substantially less likely to have access to healthcare, especially specialty services, and might lack access to some diagnostic tests and services (*34*,*35*). Similarly, we found that most missed diagnostic opportunities occurred in outpatient settings where less diagnostic testing is available than in hospital settings. Previous reports have also indicated that a large proportion of cases are diagnosed during hospital stays rather than during outpatient visits (*36*).

Patients with previous lung disease (e.g., asthma, COPD) or pulmonary imaging were also more likely to experience a missed opportunity, which is likely secondary to attributing the signs and symptoms associated with coccidioidomycosis to the patient’s previous lung disease. Indeed, missed opportunities were associated with prescriptions for inhalers, suggesting that some missed opportunities were caused by clinicians attributing symptoms related to coccidioidomycosis to reactive or obstructive airway diseases. Comparable results have been reported for other lung infections, including tuberculosis (*25*) and histoplasmosis (*23*).

Surprisingly, patients living in Arizona were at higher risk of experiencing a missed diagnostic opportunity. We propose 2 possible reasons for this finding. First, in Arizona, the harder-to-diagnose cases (e.g., mild cases with subtle symptoms) might be diagnosed, but only after longer-than-average periods of time. However, in non–coccidioidomycosis-endemic regions, harder-to-diagnose cases might go undiagnosed altogether, leaving only the cases with classic symptoms, which are easier to diagnose. As a result, the time to diagnosis might appear shorter than it would otherwise in nonendemic regions. Second, although coccidioidomycosis is much more common in Arizona than in nonendemic regions, physicians in Arizona might not prioritize coccidioidomycosis as highly in their differential diagnosis because of a form of base-rate neglect. Indeed, physicians in nonendemic regions might consider coccidioidomycosis in their differential diagnosis as soon as history of travel to an endemic region is discovered. Rapid consideration of coccidioidomycosis is based on activation of a classical illness script rather than a consideration of the actual probability of disease risk. That overconsideration of coccidioidomycosis might help to reduce diagnosed delays for this disease outside of endemic regions.

Several studies have focused on the inappropriate use of antibiotics before the diagnosis of coccidioidomycosis (*13*–*16*,*37*,*38*); most patients receive an antibiotic before diagnosis, and a substantial proportion of patients receive multiple rounds of antibiotics (*38*). In a California study (*37*), 70% of patients testing positive for coccidioidomycosis received an antibiotic before diagnosis; the median number of antibiotic prescriptions for that population was 3. Our modeling framework enabled us to explore the potential association of antibiotic prescriptions and the likelihood of experiencing a missed opportunity before the coccidioidomycosis diagnosis. We found that persons who had been prescribed an antibiotic during the delay window had >3 times the odds of experiencing a missed opportunity. That finding might suggest that diagnostic delays are a potentially unappreciated harm with respect to inappropriate antibiotic use. Similar findings have been reported for tuberculosis (*25*), histoplasmosis (*23*), pertussis (*21*), herpes encephalitis (*24*), and sepsis (*20*). Current Infectious Diseases Society of America community-acquired pneumonia guidelines are silent on testing for endemic mycoses (*39*). Therefore, it might be useful for patients not improving on an initial course of antibiotics to be reevaluated for alternative diagnoses, including endemic mycoses, given their frequency in certain regions or after travel to high-risk areas.

The first limitation of this study is that we relied on administrative claims data that are routinely collected for billing purposes but might not capture key factors related to diagnostic delays. For example, we used diagnostic codes to identify cases of coccidioidomycosis and previous symptomatic visits. We did not have access to laboratory results or radiology reports to confirm cases of disease. We also did not have access to clinical notes that could have captured signs and symptoms not assigned to a diagnosis code. Thus, some patients with coccidioidomycosis might be misclassified, and symptoms that occurred before diagnosis might have been underidentified. In addition, although we have limited geographic data (i.e., enrollee state, metropolitan statistical area for Commercial and Medicaid populations), details of travel history or localized exposures, which might be confounders in the diagnostic delay process, were unknown. Finally, the study population was restricted to commercially insured persons who were enrolled sometime during 2001–2022 and maintained continuous enrollment coverage for >1 year before diagnosis. That factor might limit generalizability to uninsured populations, persons who have frequent gaps in insurance coverage, or current and future patient populations outside the study period.

In conclusion, our study suggests that diagnostic delays in coccidioidomycosis are common and that multiple missed opportunities occur frequently. Raising clinician awareness and expanding access to timely diagnostic testing, particularly in outpatient settings, are critical steps toward improving outcomes for patients with coccidioidomycosis.

Appendix Additional information about frequency and duration of diagnostic delays associated with coccidioidomycosis and risk factors for missed diagnoses, United States.
