# Supplementary material for: Frequency and Duration of Diagnostic Delays Associated with Coccidioidomycosis and Risk Factors for Missed Diagnoses, United States
Source: Emerg Infect Dis. 2026 May;32(5):697–706. doi: 10.3201/eid3205.251421 (PMC13174966; doi:10.3201/eid3205.251421)
Supplement: Appendix — Additional information about frequency and duration of diagnostic delays associated with coccidioidomycosis and risk factors for missed diagnoses, United States. [file 25-1421-Techapp-s1.pdf]

Article DOI: https://doi.org/10.3201/eid3205.251421

EID cannot ensure accessibility for supplementary materials supplied by authors. Readers who have difficulty accessing supplementary content should contact the authors for assistance.

Frequency and Duration of Diagnostic Delays Associated with Coccidioidomycosis and Risk Factors for Missed Diagnoses, United States

Appendix

Appendix Table 1. ICD-9/10-CM codes used to identify potential missed diagnostic opportunities

ICD Code	ICD Version	Description
038.9	9	Unspecified septicemia
079.99	9	Unspecified viral infection
135	9	Sarcoidosis
162.3	9	Malignant neoplasm of upper lobe, bronchus or lung
162.5	9	Malignant neoplasm of lower lobe, bronchus or lung
162.9	9	Malignant neoplasm of bronchus and lung, unspecified
212.3	9	Benign neoplasm of bronchus and lung
235.7	9	Neoplasm of uncertain behavior of trachea, bronchus, and lung
239.1	9	Neoplasm of unspecified nature of respiratory system
276.1	9	Hyposmolality and/or hyponatremia
284.19	9	Other pancytopenia
287.5	9	Thrombocytopenia, unspecified
288.3	9	Eosinophilia
288.60	9	Leukocytosis, unspecified
322.9	9	Meningitis, unspecified
423.9	9	Unspecified disease of pericardium
428.0	9	Congestive heart failure, unspecified
429.3	9	Cardiomegaly
461.9	9	Acute sinusitis, unspecified
462	9	Acute pharyngitis
465.8	9	Acute upper respiratory infections of other multiple sites
465.9	9	Acute upper respiratory infections of unspecified site
466.0	9	Acute bronchitis
473.9	9	Unspecified sinusitis (chronic)
481	9	Pneumococcal pneumonia [Streptococcus pneumoniae pneumonia]
482.89	9	Pneumonia due to other specified bacteria
482.9	9	Bacterial pneumonia, unspecified
483.8	9	Pneumonia due to other specified organism
485	9	Bronchopneumonia, organism unspecified
486	9	Pneumonia, organism unspecified
487.1	9	Influenza with other respiratory manifestations
490	9	Bronchitis, not specified as acute or chronic
491.20	9	Obstructive chronic bronchitis without exacerbation
491.21	9	Obstructive chronic bronchitis with (acute) exacerbation
491.9	9	Unspecified chronic bronchitis
492.0	9	Emphysematous bleb
492.8	9	Other emphysema
493.00	9	Extrinsic asthma, unspecified
493.10	9	Intrinsic asthma, unspecified
493.20	9	Chronic obstructive asthma, unspecified

ICD Code	ICD Version	Description
493.22	9	Chronic obstructive asthma with (acute) exacerbation
493.90	9	Asthma, unspecified type, unspecified
493.92	9	Asthma, unspecified type, with (acute) exacerbation
496	9	Chronic airway obstruction, not elsewhere classified
511.0	9	Pleurisy without mention of effusion or current tuberculosis
511.1	9	Pleurisy with effusion, with mention of a bacterial cause other than tuberculosis
511.89	9	Other specified forms of effusion, except tuberculous
511.9	9	Unspecified pleural effusion
512.8	9	Other pneumothorax and air leak
512.81	9	Primary spontaneous pneumothorax
512.82	9	Secondary spontaneous pneumothorax
512.83	9	Chronic pneumothorax
512.84	9	Other air leak
512.89	9	Other pneumothorax
513.0	9	Abscess of lung
514	9	Pulmonary congestion and hypostasis
515	9	Postinflammatory pulmonary fibrosis
518.0	9	Pulmonary collapse
518.3	9	Pulmonary eosinophilia
518.81	9	Acute respiratory failure
518.82	9	Other pulmonary insufficiency, not elsewhere classified
518.84	9	Acute and chronic respiratory failure
518.89	9	Other diseases of lung, not elsewhere classified
519.11	9	Acute bronchospasm
695.2	9	Erythema nodosum
719.40	9	Pain in joint, site unspecified
729.1	9	Myalgia and myositis, unspecified
780.6	9	Fever and other physiologic disturbances of temperature regulation
780.60	9	Fever, unspecified
780.61	9	Fever presenting with conditions classified elsewhere
780.62	9	Postprocedural fever
780.63	9	Postvaccination fever
780.64	9	Chills (without fever)
780.65	9	Hypothermia not associated with low environmental temperature
780.66	9	Febrile nonhemolytic transfusion reaction
780.79	9	Other malaise and fatigue
780.8	9	Generalized hyperhidrosis
782.1	9	Rash and other nonspecific skin eruption
782.2	9	Localized superficial swelling, mass, or lump
783.21	9	Loss of weight
784.0	9	Headache
784.2	9	Swelling, mass, or lump in head and neck
785.0	9	Tachycardia, unspecified
785.6	9	Enlargement of lymph nodes
786.00	9	Respiratory abnormality, unspecified
786.05	9	Shortness of breath
786.07	9	Wheezing
786.09	9	Other respiratory abnormalities
786.2	9	Cough
786.3	9	Hemoptysis
786.30	9	Hemoptysis, unspecified
786.31	9	Acute idiopathic pulmonary hemorrhage in infants [AIPHI]
786.39	9	Other hemoptysis
786.50	9	Chest pain, unspecified
786.51	9	Precordial pain
786.52	9	Painful respiration
786.59	9	Other chest pain
786.6	9	Swelling, mass, or lump in chest
786.9	9	Other symptoms involving respiratory system and chest
787.20	9	Dysphagia, unspecified
787.91	9	Diarrhea
789.00	9	Abdominal pain, unspecified site
789.01	9	Abdominal pain, right upper quadrant
789.06	9	Abdominal pain, epigastric
789.07	9	Abdominal pain, generalized
789.09	9	Abdominal pain, other specified site
790.8	9	Viremia, unspecified
793.1	9	Lung field
793.11	9	Solitary pulmonary nodule

ICD Code	ICD Version	Description
793.19	9	Other nonspecific abnormal finding of lung field
799.02	9	Hypoxemia
995.91	9	Sepsis
V74.1	9	Screening examination for pulmonary tuberculosis
M25.50	10	Pain in unspecified joint
M79.1	10	Myalgia
M79.10	10	Myalgia, unspecified site
M79.11	10	Myalgia of mastication muscle
M79.12	10	Myalgia of auxiliary muscles, head and neck
M79.18	10	Myalgia, other site
R04.2	10	Hemoptysis
R05	10	Cough
R06.00	10	Dyspnea, unspecified
R06.02	10	Shortness of breath
R06.09	10	Other forms of dyspnea
R06.2	10	Wheezing
R06.89	10	Other abnormalities of breathing
R06.9	10	Unspecified abnormalities of breathing
R07.1	10	Chest pain on breathing
R07.2	10	Precordial pain
R07.81	10	Pleurodynia
R07.82	10	Intercostal pain
R07.89	10	Other chest pain
R07.9	10	Chest pain, unspecified
R10.10	10	Upper abdominal pain, unspecified
R10.11	10	Right upper quadrant pain
R10.13	10	Epigastric pain
R10.2	10	Pelvic and perineal pain
R10.30	10	Lower abdominal pain, unspecified
R10.84	10	Generalized abdominal pain
R10.9	10	Unspecified abdominal pain
R50.81	10	Fever presenting with conditions classified elsewhere
R50.9	10	Fever, unspecified
R51	10	Headache
R53.1	10	Weakness
R53.81	10	Other malaise
R53.83	10	Other fatigue
R61	10	Generalized hyperhidrosis
R63.4	10	Abnormal weight loss
A41.9	10	Sepsis, unspecified organism
B34.9	10	Viral infection, unspecified
B97.89	10	Other viral agents as the cause of diseases classified elsewhere
G03.9	10	Meningitis, unspecified
J01.90	10	Acute sinusitis, unspecified
J02.0	10	Streptococcal pharyngitis
J02.9	10	Acute pharyngitis, unspecified
J06.9	10	Acute upper respiratory infection, unspecified
J10.1	10	Influenza due to other identified influenza virus with other respiratory manifestations
J11.1	10	Influenza due to unidentified influenza virus with other respiratory manifestations
J13	10	Pneumonia due to Streptococcus pneumoniae
J15.8	10	Pneumonia due to other specified bacteria
J15.9	10	Unspecified bacterial pneumonia
J16.8	10	Pneumonia due to other specified infectious organisms
J18.0	10	Bronchopneumonia, unspecified organism
J18.1	10	Lobar pneumonia, unspecified organism
J18.2	10	Hypostatic pneumonia, unspecified organism
J18.8	10	Other pneumonia, unspecified organism
J18.9	10	Pneumonia, unspecified organism
J20.8	10	Acute bronchitis due to other specified organisms
J20.9	10	Acute bronchitis, unspecified
J32.9	10	Chronic sinusitis, unspecified
J40	10	Bronchitis, not specified as acute or chronic
J85.1	10	Abscess of lung with pneumonia
J85.2	10	Abscess of lung without pneumonia
N39.0	10	Urinary tract infection, site not specified
C34.10	10	Malignant neoplasm of upper lobe, unspecified bronchus or lung
C34.11	10	Malignant neoplasm of upper lobe, right bronchus or lung
C34.30	10	Malignant neoplasm of lower lobe, unspecified bronchus or lung
C34.90	10	Malignant neoplasm of unspecified part of unspecified bronchus or lung

ICD Code	ICD Version	Description
D14.30	10	Benign neoplasm of unspecified bronchus and lung
D38.1	10	Neoplasm of uncertain behavior of trachea, bronchus and lung
D49.1	10	Neoplasm of unspecified behavior of respiratory system
D86.0	10	Sarcoidosis of lung
D86.9	10	Sarcoidosis, unspecified
I27.89	10	Other specified pulmonary heart diseases
I31.9	10	Disease of pericardium, unspecified
I50.9	10	Heart failure, unspecified
J42	10	Unspecified chronic bronchitis
J43.9	10	Emphysema, unspecified
J44.1	10	Chronic obstructive pulmonary disease with (acute) exacerbation
J44.9	10	Chronic obstructive pulmonary disease, unspecified
J45.40	10	Moderate persistent asthma, uncomplicated
J45.901	10	Unspecified asthma with (acute) exacerbation
J45.909	10	Unspecified asthma, uncomplicated
J45.998	10	Other asthma
J80	10	Acute respiratory distress syndrome
J81.1	10	Chronic pulmonary edema
J82	10	Pulmonary eosinophilia, not elsewhere classified
J84.10	10	Pulmonary fibrosis, unspecified
J84.89	10	Other specified interstitial pulmonary diseases
J84.9	10	Interstitial pulmonary disease, unspecified
J85.0	10	Gangrene and necrosis of lung
J86.9	10	Pyothorax without fistula
J91.8	10	Pleural effusion in other conditions classified elsewhere
J93.83	10	Other pneumothorax
J94.1	10	Fibrothorax
J94.8	10	Other specified pleural conditions
J94.9	10	Pleural condition, unspecified
J96.00	10	Acute respiratory failure, unspecified whether with hypoxia or hypercapnia
J96.01	10	Acute respiratory failure with hypoxia
J96.21	10	Acute and chronic respiratory failure with hypoxia
J96.90	10	Respiratory failure, unspecified, unspecified whether with hypoxia or hypercapnia
J98.01	10	Acute bronchospasm
J98.19	10	Other pulmonary collapse
J98.4	10	Other disorders of lung
R04.9	10	Hemorrhage from respiratory passages, unspecified
R09.1	10	Pleurisy
D61.818	10	Other pancytopenia
D69.6	10	Thrombocytopenia, unspecified
D72.1	10	Eosinophilia
D72.829	10	Elevated leukocyte count, unspecified
E871	10	Hypo-osmolality and hyponatremia
I51.7	10	Cardiomegaly
J90	10	Pleural effusion, not elsewhere classified
J98.11	10	Atelectasis
R00.0	10	Tachycardia, unspecified
R09.02	10	Hypoxemia
R22.0	10	Localized swelling, mass and lump, head
R22.1	10	Localized swelling, mass and lump, neck
R22.2	10	Localized swelling, mass and lump, trunk
R22.9	10	Localized swelling, mass and lump, unspecified
R59.0	10	Localized enlarged lymph nodes
R59.1	10	Generalized enlarged lymph nodes
R59.9	10	Enlarged lymph nodes, unspecified
R74.0	10	Nonspecific elevation of levels of transaminase and lactic acid dehydrogenase [LDH]
R91.1	10	Solitary pulmonary nodule
R91.8	10	Other nonspecific abnormal finding of lung field
R94.5	10	Abnormal results of liver function studies
Z11.1	10	Encounter for screening for respiratory tuberculosis
R21	10	Rash and other nonspecific skin eruption
L52	10	Erythema nodosum

Appendix Table 2. Sensitivity analysis: impact of delay window specification

Delay Measure	Start of delay window = 84	Start of delay window = 91	Start of delay window = 98
Percent of patients that experienced a delay (95% CI)*	58.5 (95% CI: 57.0–59.8)	59.7 (95% CI: 58.3–61.1)	60.8 (95% CI: 59.1–62.3)
Mean number of missed opportunities per patient (95% CI)	2.62 (95% CI: 2.52–2.72)	2.70 (95% CI: 2.58–2.81)	2.78 (95% CI: 2.65–2.89)
Mean delay duration (days) (95% CI)	27.78 (95% CI: 26.38–29.10)	29.69 (95% CI: 28.25–31.18)	31.50 (95% CI: 29.77–33.13)

*Defined as the percent of patients that experienced at least one missed opportunity.

Appendix Table 3. Additional factors included in the exploratory risk-factor model.

Potential Risk Factor	Odds Ratio (95%CI)
Month	
January	REF
February	0.95 (0.83–1.08)
March	0.96 (0.84–1.10)
April	1.00 (0.86–1.15)
May	0.95 (0.83–1.09)
June	0.99 (0.87–1.14)
July	0.94 (0.80–1.09)
August	0.96 (0.84–1.09)
September	1.07 (0.92–1.21)
October	1.00 (0.87–1.13)
November	1.11 (0.95–1.29)
December	1.20 (1.04–1.37)
Year	
2002	REF
2003	1.20 (0.86–1.63)
2004	1.55 (1.17–2.01)
2005	1.44 (1.10–1.88)
2006	1.43 (1.06–1.86)
2007	1.45 (1.11–1.87)
2008	1.55 (1.16–2.05)
2009	2.14 (1.70–2.70)
2010	2.43 (1.84–3.05)
2011	2.42 (1.84–3.09)
2012	2.18 (1.66–2.81)
2013	2.11 (1.60–2.70)
2014	2.03 (1.54–2.61)
2015	1.71 (1.23–2.22)
2016	1.99 (1.51–2.56)
2017	1.83 (1.32–2.40)
2018	2.10 (1.55–2.86)
2019	1.95 (1.45–2.63)
2020	2.34 (1.66–3.13)
2021	2.27 (1.63–3.08)
2022	2.17 (1.51–2.87)

Appendix Table 4. Sensitivity analysis: simulation results of number and duration of delayed visits per patient by cohort*

Metric	Full Cohort (n = 26,905) Percent of patients (95%CI)†	Non-Arizona Cohort (n = 17,019) Percent of patients (95%CI)†	Arizona Cohort (n = 9,053) Percent of patients (95%CI)†
Number of missed opportunities per patient			
≥1	59.7 (58.3–61.1)	55.5 (53.6–57.3)	69.6 (66.9–71.8)
≥2	36.3 (34.6–38.1)	33.1 (30.8–35.2)	44.6 (41.0–47.8)
≥3	21.6 (20.1–23.3)	19.9 (18.0–22.1)	26.6 (23.4–29.7)
≥4	13.0 (11.7–14.3)	12.2 (10.7–13.9)	15.4 (13.0–17.8)
≥5	8.1 (7.2–9.1)	7.8 (6.7–9.1)	9.2 (7.5–10.9)
Mean number of missed opportunities per patient	2.70 (2.58–2.81)	2.70 (2.56–2.87)	2.70 (2.53–2.88)
Median number of missed opportunities per patient	2.00 (2.00–2.00)	2.00 (2.00–2.00)	2.00 (2.00–2.00)
Duration of delayed visits			
≥1 d	59.7 (58.3–61.1)	55.5 (53.6–57.3)	69.6 (66.9–71.8)
≥2 d	57.3 (55.9–58.8)	53.0 (50.9–54.7)	67.7 (64.9–69.9)
≥3 d	56.2 (54.7–57.7)	51.7 (49.7–53.5)	66.7 (63.8–68.9)
≥4 d	55.0 (53.5–56.5)	50.4 (48.3–52.2)	65.5 (62.7–67.8)
≥5 d	53.8 (52.3–55.3)	49.1 (47.0–51.0)	64.5 (61.7–66.8)
≥6 d	52.6 (51.1–54.2)	47.9 (45.8–49.7)	63.4 (60.6–65.7)
≥7 d	51.2 (49.7–52.8)	46.5 (44.3–48.4)	62.1 (59.2–64.3)
≥10 d	46.3 (44.7–47.9)	41.9 (39.7–43.9)	56.3 (53.2–58.8)
≥14 d	41.6 (39.9–43.2)	37.5 (35.2–39.5)	50.9 (47.9–53.6)
≥17 d	37.4 (35.6–39.2)	33.6 (31.2–35.7)	46.1 (42.9–48.9)
≥21 d	33.6 (31.9–35.4)	30.3 (27.9–32.4)	41.5 (38.2–44.4)
≥30 d	24.7 (22.9–26.7)	22.1 (19.7–24.4)	31.0 (27.7–34.1)
≥45 d	14.6 (12.9–16.5)	13.3 (11.0–15.5)	18.2 (15.1–21.1)
≥60 d	8.0 (6.6–9.6)	7.5 (5.6–9.1)	9.7 (7.4–12.3)
≥90 d	0.6 (0.3–0.9)	0.7 (0.3–1.0)	0.4 (0.1–0.8)
Mean delay duration (days)	29.69 (28.25–31.18)	29.01 (26.86–30.76)	31.22 (29.23–33.09)
Median delay duration (days)	24.05 (23.00–26.00)	22.95 (21.00–25.00)	26.26 (24.00–28.00)

*833 patients had missing location information. Values are % of all patients for specific cohort unless otherwise specified.
†The distribution and mean number of potential missed diagnostic opportunities each patient experienced along with the distribution and mean and median duration of diagnostic delays (in days) are presented. Potential missed diagnostic opportunities represent healthcare visits in which sign/symptoms were present, but coccidioidomycosis was not diagnosed. Delay duration was defined as the time between the earliest potential missed diagnostic opportunity a patient experienced and their index diagnosis.

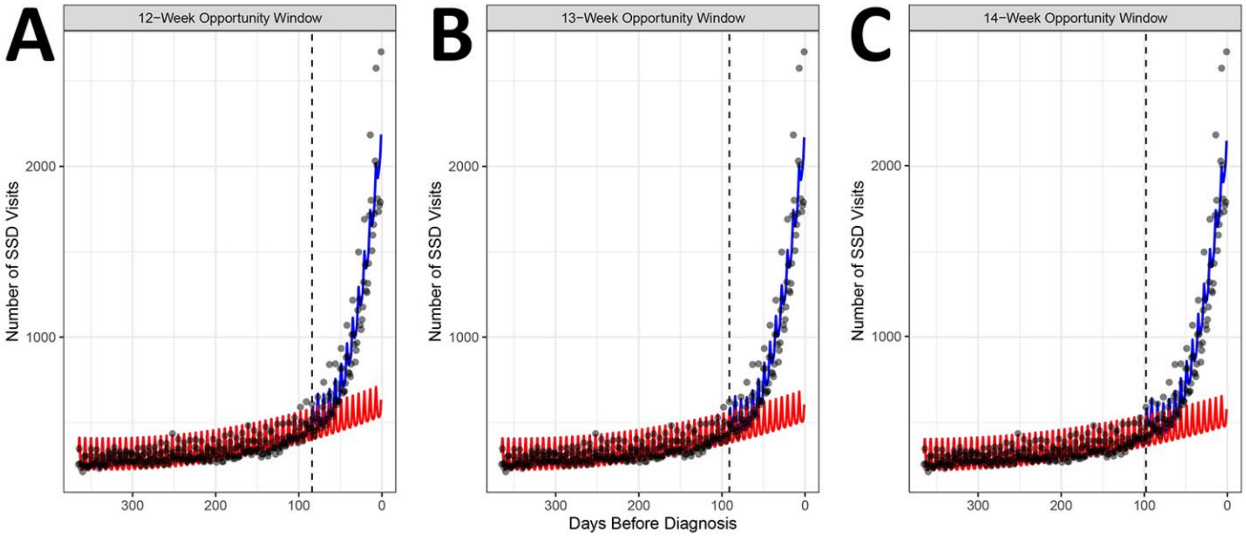

Appendix Figure 1. Comparison of expected trends in the number of SSD visits for different potential opportunity windows. The red line depicts the expected trend estimated based on the selected opportunity window. The blue line depicts the fitted trend during the diagnostic opportunity window. A 13-week window was selected as the point where the observed number of visits began to deviate from the expected trend. Opportunity windows of 12 and 14 weeks were also evaluated as part of the sensitivity analysis.

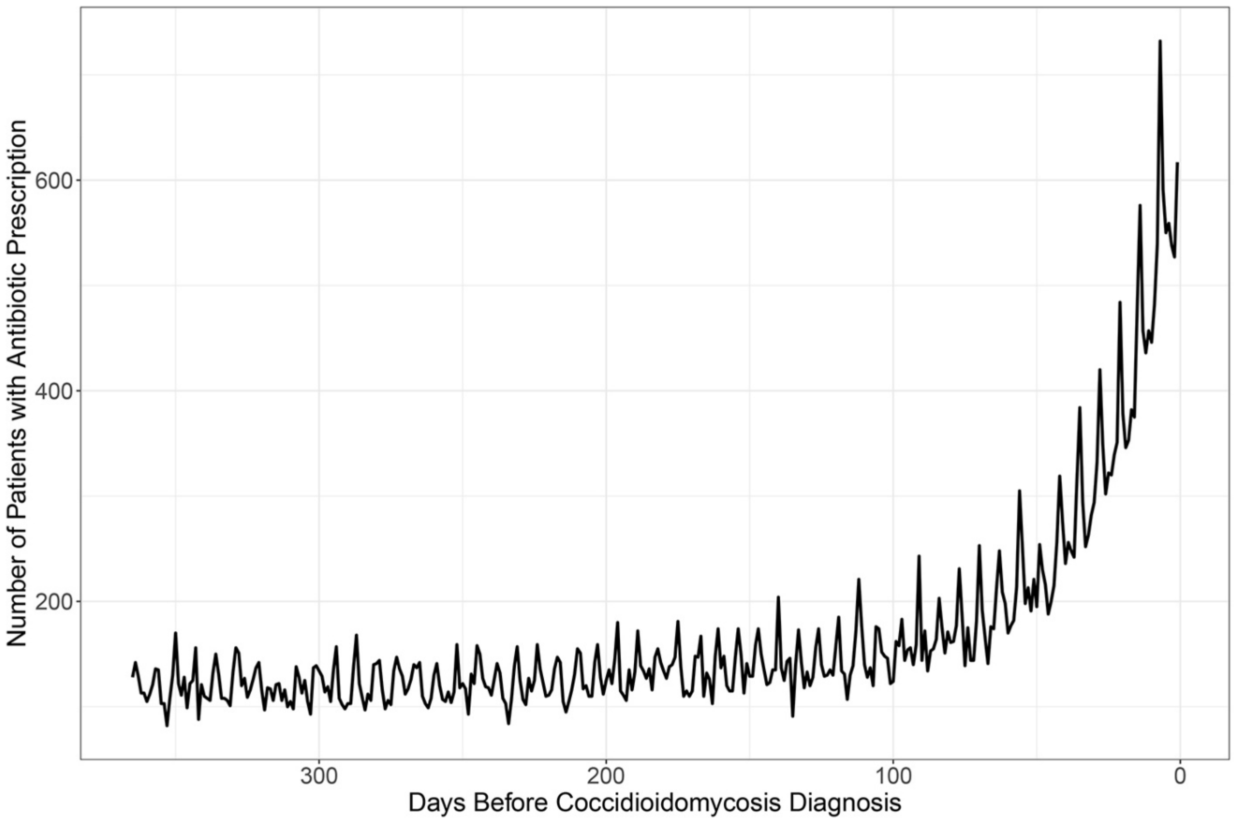

Appendix Figure 2. Number of patients with outpatient antibiotic prescription each day over the year before the index coccidioidomycosis diagnosis. There is a significant increase in outpatient antibiotic prescriptions starting around 90-days before diagnosis.
